# Haemodynamic-dependent arrest of circulating tumour cells at large blood vessel bifurcations as new model for metastasis

**DOI:** 10.1038/s41598-021-02482-x

**Published:** 2021-12-01

**Authors:** Carlos Casas-Arozamena, Alberto Otero-Cacho, Bastian Carnero, Cristina Almenglo, Maria Aymerich, Lorena Alonso-Alconada, Alba Ferreiros, Alicia Abalo, Carmen Bao-Varela, Maria Teresa Flores-Arias, Ezequiel Alvarez, Alberto P. Munuzuri, Miguel Abal

**Affiliations:** 1 Translational Medical Oncology Group (Oncomet), Health Research Institute of Santiago de Compostela (IDIS), University Hospital of Santiago de Compostela (SERGAS), Trav. Choupana s/n, 15706 Santiago de Compostela, Spain; 2 Institute CRETUS, Group of Nonlinear Physics, Department of Physics, University of Santiago de Compostela, 15782 Santiago de Compostela, Spain; 3 Photonics4Life Research Group, Applied Physics Department, Faculty of Physics, University of Santiago de Compostela, 15782 Santiago de Compostela, Spain; 4 Cardiology Group, Health Research Institute of Santiago de Compostela (IDIS), University Hospital of Santiago de Compostela (SERGAS), Trav. Choupana s/n, 15706 Santiago de Compostela, Spain; 5 Centro de Investigacion Biomedica en Red de Enfermedades Cardiovasculares (CIBERCV), Madrid, Spain; 6 Department of Pharmacology, Pharmacy and Pharmaceutical Technology, University of Santiago de Compostela, 15782 Santiago de Compostela, Spain; 7 Centro de Investigacion Biomedica en Red de Cancer (CIBERONC), Monforte de Lemos 3-5, 28029 Madrid, Spain

**Keywords:** Oncology, Fluid dynamics, Metastasis

## Abstract

Homing of circulating tumour cells (CTC) at distant sites represents a critical event in metastasis dissemination. In addition to physical entrapment, probably responsible of the majority of the homing events, the vascular system provides with geometrical factors that govern the flow biomechanics and impact on the fate of the CTC. Here we mathematically explored the distribution of velocities and the corresponding streamlines at the bifurcations of large blood vessel and characterized an area of low-velocity at the carina of bifurcation that favours the residence of CTC. In addition to this fluid physics effect, the adhesive capabilities of the CTC provide with a biological competitive advantage resulting in a marginal but systematic arrest as evidenced by dynamic in vitro recirculation in Y-microchannels and by perfusion in in vivo mice models. Our results also demonstrate that viscosity, as a main determinant of the Reynolds number that define flow biomechanics, may be modulated to limit or impair CTC accumulation at the bifurcation of blood vessels, in agreement with the apparent positive effect observed in the clinical setting by anticoagulants in advanced oncology disease.

## Introduction

Metastasis represents the most relevant and challenging clinical event in oncology, responsible for the vast majority of cancer-related deaths^1^. The presence of tumor dissemination at diagnosis or the detection of locoregional/distant metastasis indicative of progressive disease or relapse at follow-up, determines a turning point in the evolution of the patient and a clinical decision-making involving a therapeutic strategy usually based on chemotherapy and/or target therapies. Within the whole stepwise process of metastasis, the homing of circulating tumour cells (CTC) at distant sites represents a limiting step determining the efficiency of metastatic colonization^2,3^. The main mechanisms described for the arrest of tumor cells circulating in the blood vasculature include physical occlusion in small capillaries that proceed to invade and colonize this site upon adaptation to the new environment^4^. Alternatively, CTC may actively interact with the endothelium through ligand-receptor contacts via selectin adhesion molecules including rolling on the endothelial wall, tumour cell arrest and crawling, anchoring and tumour cell transmigration through the endothelial layer^5^. In parallel, the creation of pre-metastatic niches previous to the reception of the tumour cells may improve the efficiency of the homing of CTC by remodelling the receptive microenvironment^6^. From a clinical perspective, this critical and complex phase of the metastatic process offers a unique opportunity to develop specific therapeutic strategies aiming to impair the homing of the highly accessible CTC population.

To this regard, in this work we explored the mechanical and geometrical factors resulting from the anatomical structure of the vascular system and the associated hemodynamic flows^7,8^. The physical entrapment and extravasation of CTC in the microvasculature, as a principal mechanism of distant micrometastasis, is a consequence of the combined relatively low shear forces and the signalling-based communication between tumour and endothelial cells^9^. Also relevant although probably with a minor impact on the whole process of metastasis, the blood-flow-induced hemodynamic factors such as shear rates and stress gradients, as well as recirculation especially at the branches and turns of microvasculature, might play important roles in tumour cell arrest and adhesion^10^. Additionally, while mechanical trapping in arterioles and venules may be associated with rigidity of the circulating cells, the flexibility and deformability of the more aggressive metastatic cells enable them to interact with endothelial cells in response to shear stress resulting in adhesion at microvessels^11^. Otherwise, localized vorticity at the turning points of microvessel branches should support and increase tumour cell adhesion at the branching intersections^12^. In this work, we approached the particular event of tumor cell arrest related to reduced flow velocities occurring at the bifurcation of large blood vessels. The results show a limited but systematic impact of CTC homing at these particular sites that could be clinically modulated by modifying the blood flow parameters.

## Results and discussion

A numerical approach has been done to simulate and characterize the behaviour of CTC by considering a mathematical model to describe representative and physiological large blood vessel bifurcations^13^. We considered vessels of two millimetres in diameter and semi-circular cross section, inlet fluid flow of 3 mL/min and zero pressure outlet conditions; this guaranteed Reynolds numbers lower than 100 and, thus, laminar flow. CTCs (*n* = 20,000) were modelled as rigid spheres with a diameter of 14µm^14^ and a density of 1.05 g/mL^15^. The driving forces acting upon them were gravity (F_g_) and drag (F_d_), described in “Materials and methods” section, in the framework of the Schiller-Naumann method^16^ suitable for spherical solid particles. Fluid dynamics have been simulated by incompressible Navier–Stokes equations, also described in “Materials and methods” section.

**Figure 1:**
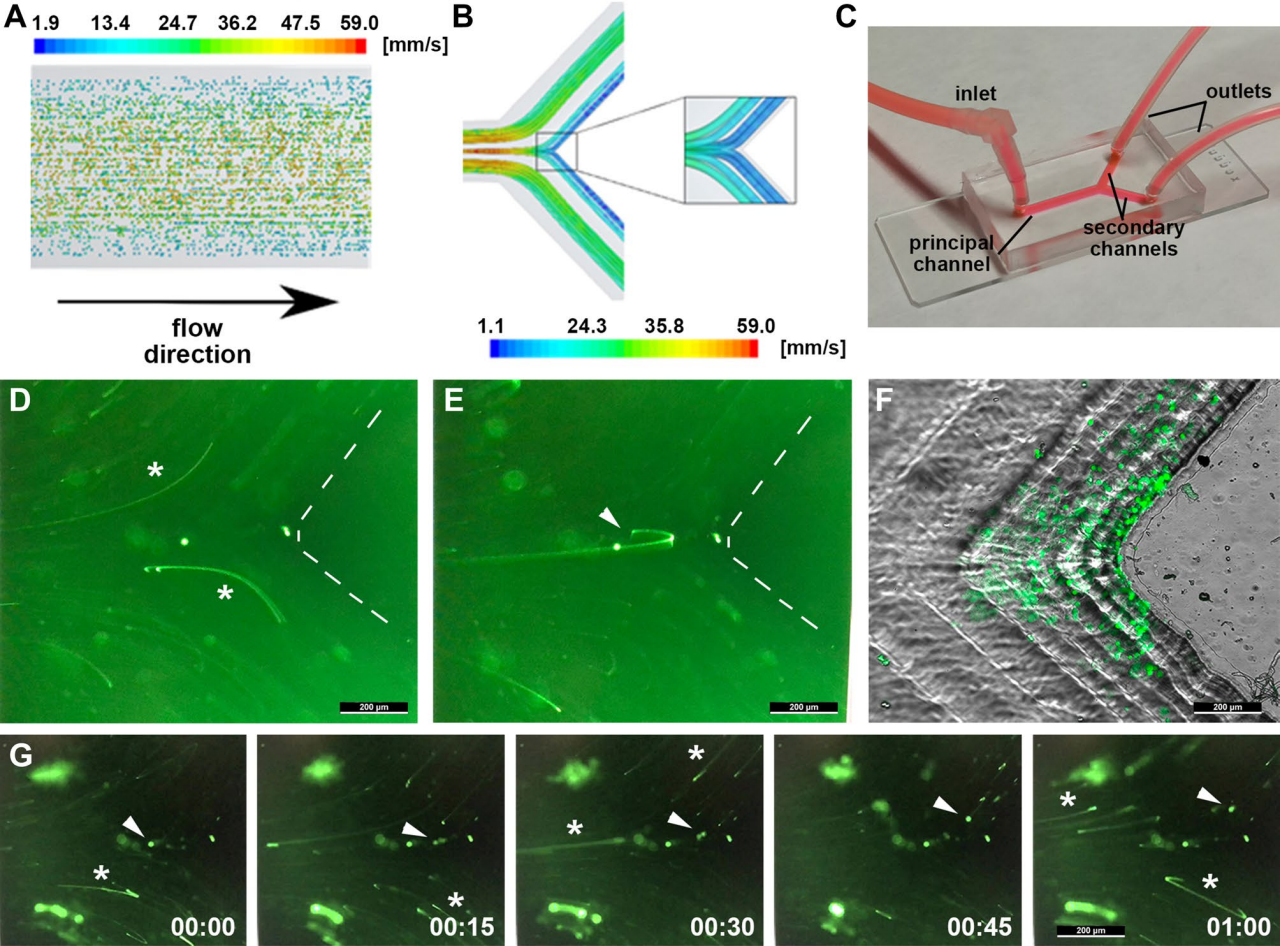
(**A**) Schematic representation of the distribution of particles and color-coded velocities in the direction of the flow. Note that the particles with highest flow velocity are located in the centre of the channel while those distributed at the walls flow more slowly. (**B**) Graphical description of the flow-lines distribution at the bifurcation of a representative blood vessel based on the numerical simulations of the mathematical model. The streamlines are color-coded following the value of the velocity in mm/s. (**C**) Image of the microfluidic Y-chamber connected to the perfusion pump for the in vitro fluidic experiments mimicking a vessel bifurcation. (**D**, **E**) Representative fluorescence images of the trajectories of GFP-tagged CTCs in the microfluidic chambers following the high-velocity streamlines from the principal towards the secondary channel (asterisks in panel **D**), and the minoritarian component following the inertial trajectories towards the low-velocity area at the carina (arrowhead in panel **E**). The vertex of the bifurcation has been delineated by dashed lines. (**F**) Arrest of GFP-tagged CTC at the low-velocity area of the bifurcation resulting from the inertial trajectories upon CTC recirculation. (**G**) Representative sequence of a GFP-tagged CTC illustrating the residence time at the carina (arrowhead), in comparison with the trails resulting from the majoritarian CTC following the high-velocity streamlines (asterisks); the entire sequence lasts one second, as numerically indicated.

Using this mathematical model and numerical simulations, we considered CTC distributed throughout the cross section of the principal vessel (Fig. 1A). At the bifurcation, the flow in the principal vessel splits into the two secondary vessels with most of the CTC being distributed following the streamlines with the maximum velocity (green lines in Fig. 1B). More interestingly, the simulations also predicted that a minor component of the CTC may leave the main streamlines due to their inertial trajectories and reach locations very close to the vessel wall at the vertex of the bifurcation (carina) with extremely low velocities (blue lines in Fig. 1B). This minor component represents around 0.1% of the total CTC trajectories and it is associated with the distribution of the CTC in the caudal of the principal vessel, those at the centre flowing with the highest velocities and thus with increased probability to reach the carina by inertial forces (Fig. 1A). At the areas of reduced velocity, the time of residence of these CTC may be relevant enough to permit the interaction with the endothelial walls and to eventually adhere.

To experimentally evaluate the behaviour of the CTC in this particular context, we fabricated microfluidic chamber by laser indirect writing^17^, with circular symmetry. In particular, to allow a better optical inspection of the trajectory of the cells, semi blood vessel-like masks were made, with a 2 mm diameter. These microfluidic chambers were then replicated by soft lithography with polydimethylsiloxane (PDMS), presenting appropriate properties such as light transmittance, oxygen permeability and biocompatibility. The channel chambers were sealed to a microscope slide by using a plasma oxygen technique, and the principal and secondary microchannels were coated with Type I Collagen as major extracellular matrix protein, at 100 µg/mL concentration. Finally, circulation of the CTC (0.5 × 10^6^ cell/mL) was replicated by perfusion of green fluorescent protein (GFP)-labelled MDA-MB-231 metastatic breast tumour cells at 3 mL/min within these channels, recirculating for up to 2 h (Fig. 1C). Both the computational studies and the in vitro studies employed comparable rates of CTC perfusion, considering the concentration of spheres and cells per mL, the flow rate and the perfusion time. Also, these rates of CTC perfusion are clinically relevant considering a mean detection of 1–10 CTC per 7.5 mL in metastatic breast cancer patients and a median survival rate of 3 years, and adapting and scaling the probability of CTC events in a precise position of the vasculature during the clinical history of these patients to a laboratory assay. As observed in the representative images capturing the GFP-fluorescent trajectories of the perfused CTC, behaving in concordance with the theorical prediction, the vast majority flowed into the secondary channels following the mainstream flow lines (see representative trajectories by GFP-fluorescent wakes left by flowing CTC in Fig. 1D, and complete video acquisition in Supplementary Material) and did not present any arrest or homing at this level of the vasculature, further circulating towards hypothetic capillaries and microvessels. Also as predicted by the numerical simulations, a marginal component of the CTC in the principal channel continued flowing straight towards the carina, and reached the area characterized by a reduced flow velocity (Fig. 1E; Supplementary Video S1). Upon recirculation, the microchannels were further perfused with phosphate buffer solution (PBS) for 5 min to remove any floating CTC remaining in circulation, and cells adhered to the collagen coating were fixed with paraformaldehyde (PFA, 4%). Of importance, the accumulated impacts of the CTC at the carina resulted in a systematic arrest of tumour cells at the area in front of the carina (Fig. 1F). The quantification of CTC arrested at the carina of the bifurcations resulted in a mean 85 ± 10 (mean ± SD) cells; considering the number of cells per mL, the flow rate and the duration of the perfusion, around a 0,0005% of the recirculated CTC arrested at this specific area of the simulated large blood vessels. This indicates that a small percentage of tumor cells approaching the bifurcation are eventually arrested, suggesting that the time of residence at this area of low velocity is critical for the CTC to interact with the surface of the channels. The representative sequence of an individual CTC inertially flowed towards the vertex of the bifurcation, illustrates its residence at this area of low velocities for a relevant duration (white arrow in Fig. 1G), in comparison with the majoritarian mainstream lines.

**Figure 2:**
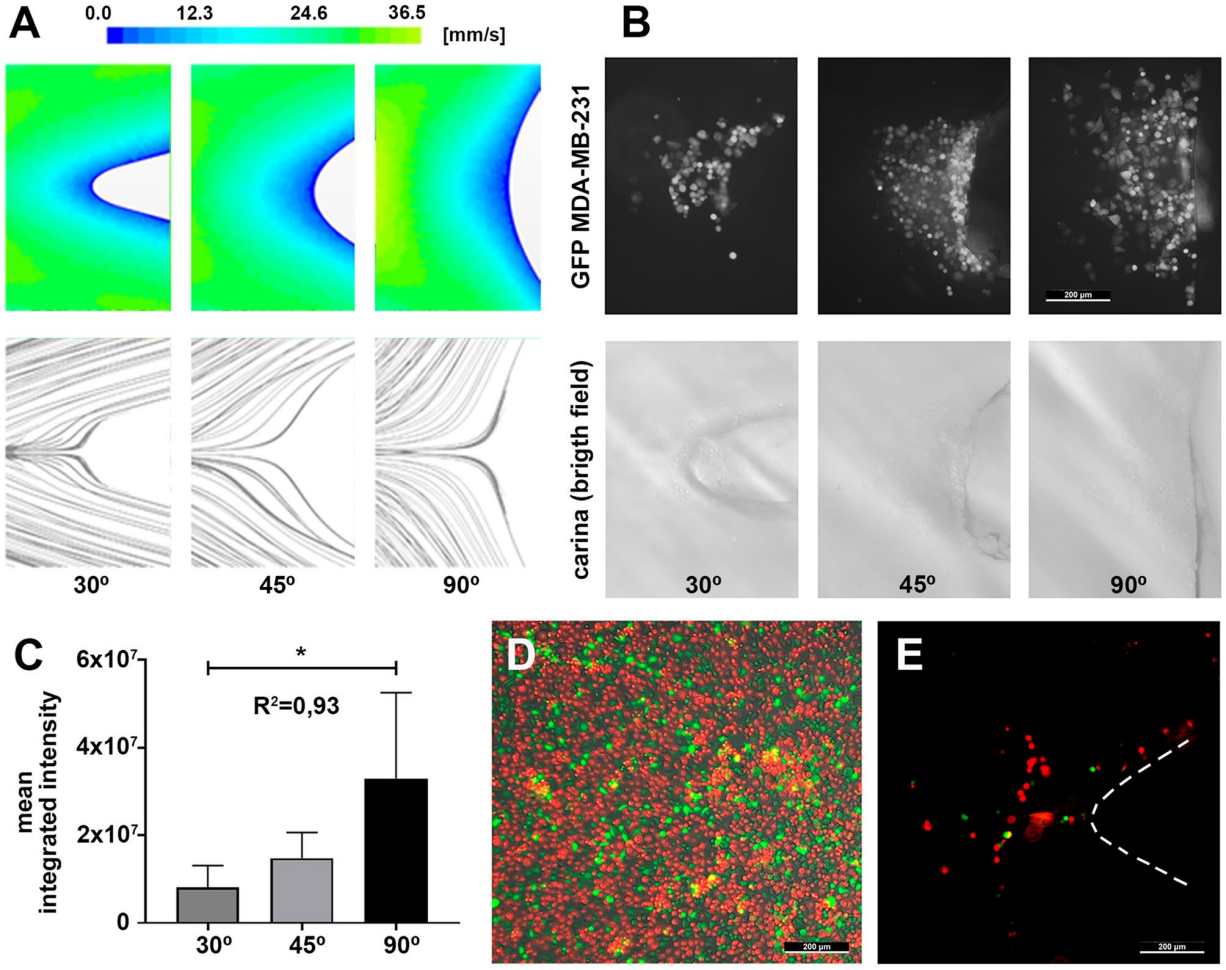
(**A**) Simulations illustrating the distribution of areas of color-coded flow velocities depending on the angle of the bifurcation (30°, 45° and 90°). Note that as the bifurcation angle increases, the area at the carina characterized by an extremely low velocity becomes larger (upper coloured row), and the streamlines followed by the simulated CTC result in larger residence times for each configuration (lower black and white row). (**B**) Representative fluorescence GFP-tagged CTC images (upper row), illustrating the concordant CTC arrest at the carina as the angle of the bifurcation increases as represented in the bright field panels (lower row); the size bar is valid for all the images. (**C**) Quantification of CTC arrest calculated by integrated intensity, is graphically described (*t*-test *p* > 0.05; R^2^ = 0.93). (**D**) Representative fluorescence image of red-tagged TIMP1 silenced MDA-MB-231 and green-tagged wild-type TIMP1 MDA-MB-231 cells adhered to the bottom of a plastic well under static conditions, illustrating the improved CTC adhesive properties upon TIMP1 silencing. (**E**) Representative fluorescence image of the red-tagged TIMP1 silenced MDA-MB-231 and green-tagged wild-type TIMP1 MDA-MB-231 cells deposited at the carina of the microchannel after competitive recirculation, illustrating the privileged arrest of the improved adhesive CTC with silenced TIMP1.

The causality of this observation was further confirmed by modifying the angle of the bifurcation. As shown in the numerical simulations, the area proximate to the vertex of the bifurcation defined by a reduced flow velocity is directly dependent on the surface of the carina that is exposed to the inertial lines of the flow and should impact on the number of CTC accessing this area and on their time of residence (Fig. 2A). Concordantly, the experimental in vitro microfluidic model comprising the perfused MDA-MB-231 metastatic cells recirculating into the PDMS channels fabricated with bifurcations angles of 30°, 45° and 90° resulted in an increased arrest of the CTC as the angle increases (Fig. 2B). The quantification of the CTC arrest confirmed the increment of this event as the angle of the bifurcations and, consequently, the area of low flow rate augment (Fig. 2C). All this supports the specificity of the low-velocity area event in the proximity of large vessel bifurcations impacting on CTC fate, and excludes an artefactual observation related to the numerical and experimental conditions. It must be considered that biomechanics constrains within the microcirculation can tune the metastatic potential of the CTC in terms of morphological features and gene expression that may affect their fate and aggressiveness^18^. The magnitude of the low-velocity area at the bifurcation is also dependent on the blood flow, affecting the Reynolds number, with very low flow rates associated with large areas of reduced velocity that may result in a prolonged residence of the CTC and an increased homing (Supplementary Fig. S1A). Also related, the wall shear stress associated with the physiological flow rates analysed may impact on the adhesive properties of the CTC^19^ (Supplementary Fig. S1B).

We next questioned whether the peripheral blood mononuclear cells (PBMC) component, with comparable dimensions and densities as the CTC but with a significantly higher proportion in circulation, behaved similarly to tumour cells at the vertices of the vessel bifurcations. For this, PBMC were purified by differential density centrifugation in Ficoll, labelled with the lipidic DiD fluorescent dye, and recirculated in the microchannels as described for the CTC. Although the access of the PBMC to the low velocity area in the proximity of the carina could be observed at similar rates as for the CTC, no PBMC arrest was found after 2 h of perfusion in the microfluidic chambers (Supplementary Fig. S2A). Likewise, the concomitant recirculation of both PBMC and CTC at physiological percentages in the microchannels did not preclude the arrest of CTC at the low-velocity areas of the carina (Supplementary Fig. S2B). These results are compatible with the physiological absence of PBMC adhesion and arrest at the bifurcations of large blood vessels that would be life threatening. They also suggested that in addition to the low velocity at the vertices of the bifurcations resulting in critical time lapses for the circulating cells to interact with the surface of the microchannels, these cells must own the ability to biologically interact with the ECM coating at these specific areas with reduced flow. Concordantly, the adhesive properties of PBMC to vessel walls seems to be governed by chemotactic signals coming from activated endothelial cells^20,21^, different from those participating in the process of metastasis^22^. To confirm this observation, we modified the adhesive capabilities of the MDA-MB-231 cells by stably knocking down of the tissue inhibitor of metalloproteinases 1 (TIMP1). TIMP1 expression in CTC purified from triple-negative breast cancer patients has been associated with an aggressive plasticity phenotype resulting in an increased metastatic potential^23^. More precisely, TIMP1 has been described to play a role in cell adhesion, and its silencing by lentiviral shRNA infection resulted in MDA-MB-231 cells with improved adhesion capabilities to a well-plate cell culture surface coated with collagen in static conditions (78,4% MDA-MB-231 shTIMP1 cells calculated using the percentage of covered area by the CTC as measured by fluorescence signal; red-tagged cells in Fig. 2D), as compared to the adhesive properties of the wild-type TIMP1 expressing MDA-MB-231 cells (21,6% MDA-MB-231 cells; green-tagged cells in Fig. 2D). The competitive CTC perfusion assay including equal number of both red-tagged TIMP1 silenced MDA-MB-231 and green-tagged wild-type TIMP1 MDA-MB-231 cells recirculating for 2 h in the microfluidic chambers, demonstrated the preferential arrest of the most adhesive CTC at the low-velocity area proximate to the 90° bifurcation (75.8% MDA-MB-231 shTIMP1 cells *versus* 24.2% MDA-MB-231 cells; Fig. 2E). These results confirmed that, in addition to the physical component resulting from the inertial trajectories of the cells accessing the vertices of the bifurcation with a reduced flow area, the biological component of the adhesive properties of the CTC are critical for the homing of this specific type of tumour cells faced to the majoritarian and non-adhesive hematopoietic cells.

**Figure 3:**
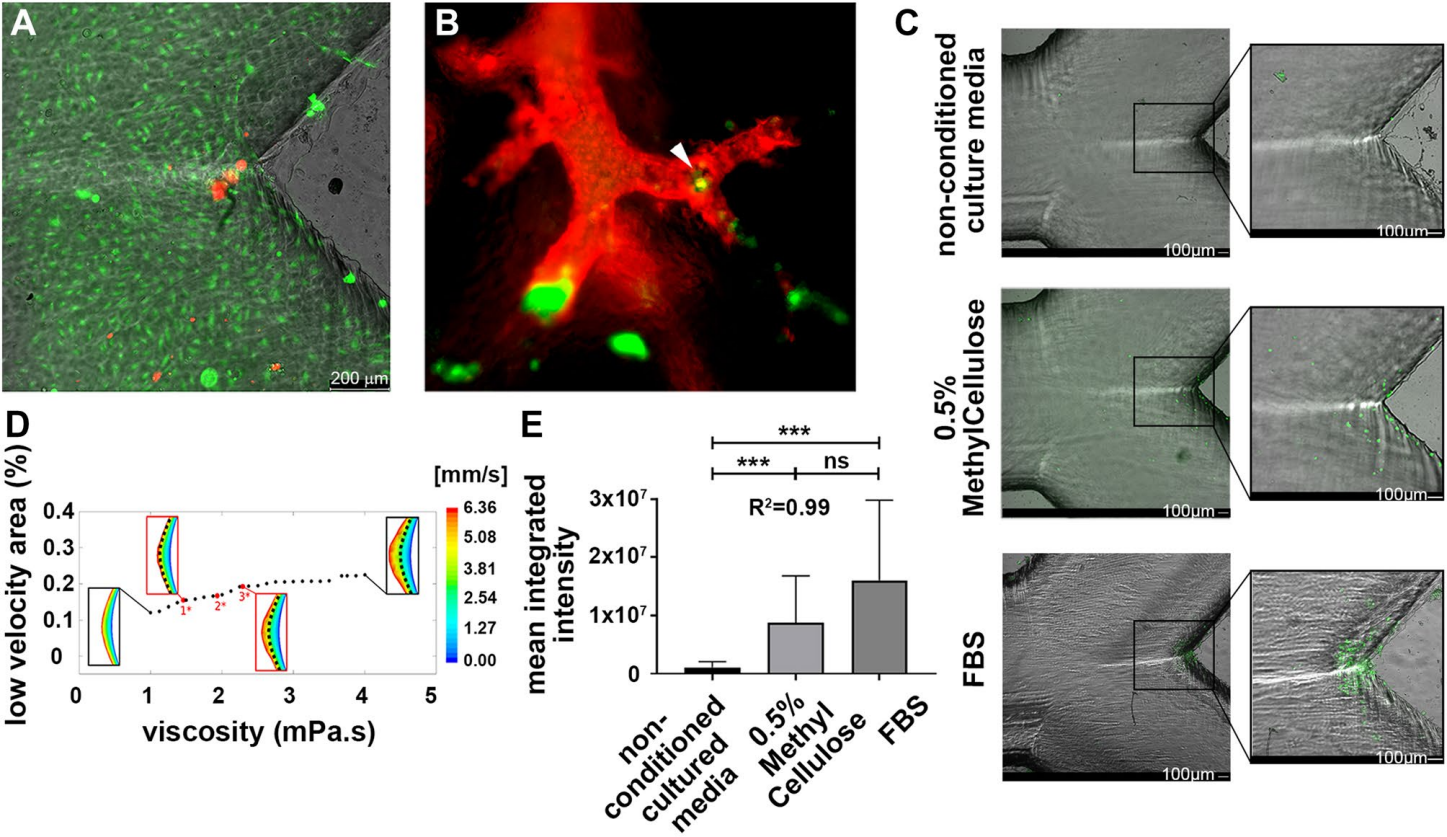
(**A**) DiD-labelled MDA-MB-231 cell (red CTC) arrest at the bifurcation of the Y-microchannel covered with calcein-labelled primary HUVEC cells (green endothelial monolayer). (**B**) Representative image of GFP-labelled MDA-MB-231 cells (green CTC) perfused in mice before direct labelling of the vasculature of the lung with DiI dye (red blood vessels), illustrating the CTC arrest at the carina of vessel bifurcations (arrowhead) in an in vivo preclinical model. The size bar in panel A is valid also for panel B. (**C**) GFP-labelled MDA-MB-231 cells embedded in three different types of fluids: non-conditioned basal culture media (upper panels), 0.5% Methylcellulose (middle panels), and FBS (foetal bovine serum; lower panels), showing improved CTC arrest at the low-velocity areas of the carina as the viscosity of the medium is increased. (**D**) Numerical representation of the low-velocity areas at the carina of the bifurcations depending on the viscosity of the medium, as calculated in the simulations. The insets correspond to the graphical representation of the areas of low velocity at different viscosity values illustrating the enlarged area as the viscosity is increased (the dashed black line marks the boundary for the lowest viscosity). The viscosity of the three different media included in panel (**C**) were numerically checked and their values (1*, non-conditioned culture medium; 2*, FBS (foetal bovine serum); and 3*, 0.5% Methylcellulose) and corresponding graphical low-velocity areas are plotted in red. (**E**) Quantification of CTC arrest calculated by integrated intensity, is graphically described. Statistical differences were found between the groups, seeing higher levels of CTC arrest with higher viscosity levels (*t*-test, ***p < 0.001; R^2^ = 0.99).

Once we described the CTC arrest singularity at the carina of the blood vessel bifurcations resulting from these marginal but relevant inertial trajectories and the adhesive capabilities of metastatic tumour cells at the reduced velocity flow areas, we explored whether these events are compatible with more complex in vitro and in vivo preclinical models. For this, we first reproduced in vitro an endothelial monolayer covering the surface of the microfluidic chambers by seeding 1.5 × 10^6^ primary human umbilical vein endothelial cells (HUVEC) onto the channels pre-treated with collagen and fibronectin for 24 h (Supplementary Video S2). Endothelial cells were labelled with calcein before recirculation of DiD-fluorescence labelled MDA-MB-231 cells for 2 h at 1.5 mL/min, to avoid the fragmentation of the endothelial layer. As shown in Fig. 3A, CTC arrest at the carina covered by the endothelial monolayer was evidenced, suggesting the formation of cell–cell contacts with the endothelial cells during the lapse time of CTC stay at the low velocity area generated at the carina of the large blood vessel bifurcations. High-resolution imaging in zebrafish embryos already demonstrated not only the relevance of blood flow in the arrest, adhesion and extravasation of tumor cells^24^, but also in the remodelling of the endothelium for further extravasation^25^. These results are also indicative of the reproducibility of the CTC close-to-the-border trajectories and homing event in a more clinically relevant scenario. Secondly, we simulated in vivo the circulation of CTC by intracardiac perfusion of GFP-labelled MDA-MB-231 cells, as described^26^. Briefly, the mice were connected to a perfusion pump by insertion of the inlet needle into the left ventricle before puncture of the right atrium. After washing with DPBS, 10 × 10^6^ CTC were allowed to recirculate through the whole vascular system at 1 mL/min during 5 min, and direct labelling of the blood vessels was achieved by perfusion of DiI, a 597 nm exciting wavelength dye, for 5 additional min. Upon perfusion, the lungs as target organ for CTC arrest and homing were surgically removed and fixed in PFA before tissue slicing in 1-mm-thick sections for direct microscopy examination. This protocol permitted the qualitative exploration of the whole organ and the confirmation of CTC arrest at the bifurcations of the large blood vessels in addition to the CTC being trapped in the alveolae, as evidenced by fluorescent microscopy (Fig. 3B). These results suggest that the homing of circulating tumour cells due to the inertial trajectories at areas of reduced flow velocity generated at the vertex of large blood vessel bifurcations that might result in the generation of distant metastasis is compatible with preclinical models that reproduce the metastatic dissemination. Simulation of cancer cell trajectories in a high-resolution humanoid model of global blood circulation combined with metastasis locations in autopsies, calculated that up to 40% of metastasis distribution may be contributed by mechanical and geometrical effects related to the blood circulation^8^.

We finally investigated whether the modulation of those components that participate in the definition and behaviour of the blood flow could minimize the contribution of the inertial homing of CTC at the bifurcations that may be in part underlying the generation of distant metastasis. For this, we modified the viscosity of the fluid as the main variable defining the Reynolds number that determines the fate of tumour cells in circulation, and compared the homing of recirculating GFP-labelled MDA-MB-231 cells in: (1) basal culture medium without foetal bovine serum (FBS) as the basic medium condition with the lowest viscosity (1.46 mPa·s); (2) FBS as the medium condition with the highest viscosity (1.935 mPa·s); and (3) basal culture medium complemented with 0.5% methylcellulose as a non-biological component adding viscosity to the medium (2.29 mPa·s), in a similar range as the FBS. The microchannels in all conditions were pre-treated with FBS previous to recirculation of the CTC, to avoid any contribution of the protein and matrix components of the FBS. As shown, CTC perfused in non-conditioned culture media with the smallest viscosity were found to arrest less efficiently at the carina of the microchannels (upper panels in Fig. 3C), compared to those perfused in culture media supplemented with 0.5% of Methylcellulose (middle panels in Fig. 3C), or to the CTC recirculating in FBS (lower panels in Fig. 3C). Concordantly, the numerical simulations of the mathematical model showed an enlargement of the areas with reduced velocities as the viscosity of the fluid is augmented (Fig. 3D), increasing the time of residence by 15% in the case of 0.5% methylcellulose or FBS compared to the non-conditioned medium fluid, and favouring the arrest of the CTC. Intriguingly, and as shown by fluorescence quantification of the CTC at the bifurcation of the channels under the three different conditions (Fig. 3E), although no statistical significance was achieved CTC arrest was found to be more efficient in FBS condition compared to 0.5% Methylcellulose, with similar but lower viscosity value, suggesting that in addition to the physical components of the fluid and the adhesive capabilities of the CTC, other biological factors may be affecting CTC homing at large blood vessel bifurcations, like CTC aggregation or clustering upon adhesion^27^.

These results, in addition to confirm the impact of flow dynamics on CTC arrest at low-velocity areas as the ones generated at large blood vessel bifurcations, also offer the opportunity to clinically impair these events as viscosity in blood is mainly governed by haematocrit and by coagulation homeostasis. Interestingly, variations in the haematocrit can modulate the adhesion of CTC to the vessel wall, their velocity in the blood flow and red blood cell aggregation^28^. Similarly, factors of the coagulation cascade, as factor V, have demonstrated a role in the interaction of the CTC with the vascular endothelium and the efficiency of metastasis^29^. From a therapeutic perspective, anticoagulant drugs as low molecular weight heparin and warfarin, contributing to reduce blood viscosity, have shown antitumor and antimetastatic activity^30,31^. In addition to viscosity, the potential anti-metastatic effects include the modulation of growth factors and anticoagulant activity, and the inhibition of selectin-mediated interactions of tumour cells with leukocytes, platelets and endothelial cells mediating the hematogenous metastasis^32^. In fact, and although there exists limited data on the effect of these drugs diminishing the occurrence of thromboembolic events, clinical studies showed a benefit in survival with an effective inhibition of the metastatic cascade rather than impacting on primary tumours^33^. Likewise, there is a considerable body of pre-clinical, epidemiological and randomized data to support the hypothesis that aspirin has the potential to be an effective adjuvant cancer therapy^34^. As above, the mechanisms underlaying the effect of aspirin on blood flow and viscosity, seem overlapped by the impact on microenvironment-centred mechanisms reducing metastasis through the inhibition of platelet COX-1^35^. The Add-Aspirin trial investigates whether regular aspirin use after standard therapy prevents recurrence and prolongs survival in participants with four non-metastatic common solid tumours (http://www.addaspirintrial.org). Similarly, direct oral anticoagulants seem to have an impact on metastasis dissemination in preclinical models^36^.

In conclusion, we here described a novel mechanism of CTC arrest at the vertex of blood vessel bifurcations, associated with low flow velocities and inertial trajectories of the tumour cells. Adhesive properties of the CTC also play a critical role as residence of the CTC in the low-velocity areas must allow the establishment of cell contacts with the endothelial layer and the ECM. Finally, we present data that support the reduction of blood viscosity to minimize/impair the critical CTC homing step in the process of metastatic colonization. The combined use of numerical simulations techniques with organ-on-a-chip technologies allowed us to complement the results and show a more insightful explanation of the mechanism.

## Materials and methods

### Numerical methods

#### Geometry and fluid dynamics

Star-CCM + software was used to design geometries, build a grid and carry-on numerical simulations. Segregated Flow solver and finite volume method (FVM) were used in order to solve the fluid-dynamic equations^37,38^. Fluid dynamics has been simulated by incompressible Navier–Stokes equations described below:

1 $$\Delta \vec{v} = 0$$

2 $$\rho \left( {\vec{v} \cdot \nabla } \right)\vec{v} = - \nabla p + \mu \Delta \vec{v}$$

where $\rho$ is fluid density, $\mu$ fluid viscosity, $\vec{v}$ fluid velocity and $p$ pressure.

Computations were run until a steady state was reached and convergence monitors were set in 1e-5.

The domain used to perform the numerical simulation was a bifurcation with different opening angles α (30°, 45° and 90°) and different vertex shape. The channel section has, in all configurations considered, a semi-circular shape with a 2 mm diameter. Details and characteristic distances of these configurations are observed in Supplementary Fig. S3.

#### Analysis of mesh independence

Numeric mesh was build using polyhedral mesh refined near wall with hexahedral layers to improve calculation accuracy. In order to ensure that the results obtained do not depend on the discretization of the geometry, the value of maximum velocity was studied for 3 different types of mesh in every geometry. All the simulations were repeated for the three different grids. In Supplementary Table S1, the specifications of each grid are summarized for the 90° geometry and sharpest bifurcation angle. The maximum value of the velocity of the fluid flow was chosen as the control variable for its significance in the problems analysed. The circulating fluid considered for this test is foetal bovine serum (FBS). Supplementary Table S2 presents a mesh study carried out for a geometry with a blunt or non-sharp bifurcation angle and taking residence time as a control variable. Note that the variations in the magnitude ‘Maximum velocity’ and ‘Residence Time’ were always less than 5%. For this reason, Mesh 1 was chosen for the simulations presented in the main text as they required less computational effort.

#### Flow

Different types of fluids have been considered both in experiments and in numerical simulations. A fluid flow of 3 mL/min was used as inlet condition for every simulation and zero pressure was set at the outlets. Reynolds number was always less than 100 and it varied only as a function of the viscosity and density since the inlet velocity and diameter were constant. Thus, laminar flow was guaranteed for all the simulations. Although the behaviour of the experimental fluids considered were non-Newtonian as we can see in Supplementary Fig. S4, in the simulations we considered a Newtonian fluid following the simplification widely used in literature. Supplementary Table S3 presents the values of the density and the viscosity (for the particular shear rates used in in-vivo experiments) for the different fluids considered experimentally.

#### Particles

20,000 CTCs were modelled as rigid spheres with a diameter of 14 µm^14^ and density 1.05 g/mL^15^. They were introduced into the fluidic domain in order to study their behaviour under flow conditions.

The forces that act on the particles are drag force and gravity and are defined below^37^:

##### Drag force

3 $$F_{d} = \frac{1}{2}C_{d} \rho A_{p} \left| {v_{s} } \right|v_{s}$$

where $C_{d}$ is the drag coefficient of the particle, $\rho$ is the density of the continuous phase, $v_{s} = v - v_{s}$ is the particle slip velocity with v being the instantaneous velocity of the continuous phase and $A_{p}$ is the projected area of the particle. Schiller-Naumann was chosen as the method to define the drag coefficient because it is suitable for spherical solid particles. Since the Particle Reynolds number is always less than 1, the drag coefficient is defined as follows:

4 $$C_{d} = \frac{24}{{Re_{p} }}\left( {1 + 0.15Re_{p}^{0.687} } \right)$$

where:

5 $$Re_{p} = \frac{{\rho \left| {v_{s} } \right|D_{p} }}{\mu }$$

with $D_{p}$ the particle diameter and $\mu$ the dynamic viscosity.

##### Gravity

6 $$F_{g} = m_{p} *g$$

with $m_{p}$ the particle mass and *g* the gravitational acceleration vector.

### Fabrication method of the fluidic device

The fluidic device for performing in vitro experiments was a concave Y-shaped channel made from polydimethylsiloxane (PDMS, Sylgard 184 from Dow Corning). In particular, to allow a better optical inspection, semi blood vessel-like devices have been fabricated. The first mould was fabricated by indirect laser writing technique (LIPAA) in a soda-lime glass substrate. The used laser was a Nd:YAG laser (Rofin PowerLine E) working at a wavelength of 1064 nm with a pulse duration of 20 ns. The parameters used of the laser were: 667 mJ energy per pulse, repetition rate of 12 kHz and a scan speed of 1000 mm/s. The hatching was 25 µm. The master was replicated in a second step filling the structure with silicone to obtain an inverse mould^17^. The silicone master was replicated by soft-lithography in PDMS. A mixed of 10:1 of PDMS was prepared for the replication. The silicone master was completely covered with this mixture. To remove all the bubbles in the PDMS, it was introduced in a vacuum chamber 40 min at 400 mbar. The silicone master covered with the PDMS was cured at a furnace for 2 h at 40 °C. The silicone was peeled out from the PDMS. This final device was bonded by the oxygen plasma technique with a Diener Zepto plasma cleaner. As described, oxygen plasma procedure transforms the hydrophobic surface of the PDMS, with a surface energy around 20–30 mJ m^−2^, into a biocompatible hydrophilic surface of around 60–80 mJ m^−2^, that improves cell adhesion^39^. This biocompatibility resulting from the moisturized surface of the channels was further enhanced with the collagen biocoating, both for CTC arrest as well as for endothelial monolayer formation (see below). A final thermal treatment was applied to improve the optical quality of the device for microscope inspection. The sealed fluidic device is introduced into a furnace for 20 min at 100 °C (Supplementary Fig. S5).

### Cell culture

The MDA-MB-231, green fluorescent protein (GFP)-labelled MDA-MB-231 and MDA-MB-231 shTIMP1 cells^23^ were maintained using Dulbecco’s Modified Eagle Medium (DMEM) (Lonza, Walkersville, MD, USA) supplemented with 10% FBS (Gibco, Grand Island, NY, USA) and 1% penicillin–streptomycin (Gibco, Grand Island, NY, USA) in a humidified atmosphere at 37 °C and 5% CO_2_. Cells lines were subcultured twice a week when an 80% confluency was reached. Briefly, the culture medium was aspirated, and the dish (Corning, New York, NY, USA) was washed with Dulbecco’s Phosphate Buffered Saline (DPBS) (Sigma-Aldrich, St. Louis, MO, USA) then cells were detached using Trypsin–EDTA (Lonza, Walkersville, MD, USA).

Human umbilical vein endothelial cells (HUVEC) were isolated from freshly obtained human umbilical cords donated under written informed consent from mothers, and following the method previously described^40^. All the procedures were approved by the Ethics Committee for Clinical Research at Galicia, according to the World Medical Association Declaration of Helsinki. Briefly, HUVEC were cultured on 0.2% (w/v) gelatine (Sigma-Aldrich; Merck Life Science S.L.U., Madrid, Spain) pre-coated flasks or dishes (Corning, New York, NY, USA) and grown in complete EGM-2 media (Endothelial Growth Medium-2, Lonza, Basel, Switzerland), containing 2% FBS between other components, in a humidity-saturated atmosphere with 5% CO_2_ at 37 °C. Cells for the experiments were used between the second and seventh passages.

### In vitro CTC perfusion assay

In all experiments the microfluidic chambers were coated with Type I collagen from rat tail (Sigma-Aldrich, St. Louis, MO, USA) at 100 µg/mL for 20 min at room temperature. Washed once with DPBS and cells were recirculated at a physiological 3 ml/min velocity for up to 2 h. Afterwards, the microfluidic chambers were washed with DPBS for 5 min to remove any remaining CTC in circulation. The adhered cells were fixed using paraformaldehyde (PFA) 4% (Ted Pella, INC., Redding, CA, USA) for 10 min and then washed with PBS. Microfluidic chambers were analysed using a LEICA DMi8 fluorescence microscope (Leica Microsystems, Wetzlar, Germany). The integrated intensity measured by ImageJ (Bethesda, Maryland, USA) analysis was used to quantify the number of CTCs arrested at the bifurcation. Mann Whitney test was used to determine the statistical relationship between the different conditions. A *p*-value ≤ 0.05 was set as the level of significance.

### PBMC assay

First peripheral blood mononuclear cells (PBMC) were isolated by density centrifugation. Briefly, blood from healthy voluntary donors under written informed consent was collected via venepuncture in EDTA tubes and mixed with PBS in a 1:1 ratio. The same amount of lymphocytes isolation solution (StemCell, Oslo, Norway) was added to the bottom of a 50 mL conical tube and the blood-DPBS mixture was carefully placed on top. Sample was centrifuged at 1200 rpm for 30 min without brake or acceleration and the peripheral blood mononuclear cells (PBMC) were separated and washed twice with DPBS. PBMC were labelled using the Vybrant DiD cell-labelling solution (Life Technologies, Eugene, OR, USA) according to the manufacturer instructions. Briefly, 5µL of DiD was added for each 1 × 10^6^ cells and incubated for 20 min at 37 °C then washed three times with DPBS. Perfusion of PBMCs and GFP-labelled MDA-MB-231 cells was done at a 10:1 ratio as previously described.

### Adhesion assay

The MDA-MB-231 shTIMP1 cells were labelled using the Vybrant DiD cell-labelling solution (Life Technologies, Eugene, OR, USA) as previously described. Red tagged TIMP1 silenced MDA-MB-231 cells and GFP-labelled MDA-MB-231 cells were perfused at a 1:1 ratio as previously described.

### Endothelial assay

For the experiments, HUVEC at confluence were detached with trypsin (0.25% in Hank’s balanced salt solution with 1 mM EDTA; Gibco, Thermo Fisher Scientific, Waltham, MA, USA) and seeded in fibronectin (5 µg/ml in 0.02% gelatine; Gibco) pre-coated PDMS channels at a concentration of 1.5 × 10^6^ cells/ml. HUVEC were labelled with calcein-AM (Invitrogen, Thermo Fisher Scientific, Waltham, MA, USA) at a 1 µM concentration for 20 min. Then, cells were rinsed twice to remove unlabelled calcein-AM; Supplementary Video S2 shows a representative 3D confocal microscopy reconstruction of the whole microchannel coated with calcein-labelled HUVEC cells. MDA-MB-231 cells were labelled using the Vybrant DiD cell-labelling solution (Life Technologies, Eugene, OR, USA) as previously mentioned. Perfusion started at 0.5 mL/min to avoid damage to the monolayer, velocity was doubled every 15 min until reaching 1.5 mL/min for 2 h.

### Viscosity assay

GFP-labelled MDA-MB-231 cells were resuspended in 20 mL of either non-conditioned medium, 0.5% hydroxypropyl methylcellulose (Sigma-Aldrich, St. Louis, MO, USA) supplemented medium, or 100% FBS. In order to minimize the impact of any eventual deposition of matrix components from the FBS on the channels, a preconditioning with FBS circulating in the microfluidic chambers for 1 h was performed previous to the perfusion of the CTC in all conditions as previously described.

### In vivo CTC perfusion assay

For the intracardiac perfusion, mice were housed and maintained under specific-pathogen-free conditions, and procedures were performed in accordance with institutional guidelines and approved by the Use Committee for Animal Care from the Universidad de Santiago de Compostela. Aseptic procedures were followed for all surgeries. The 8-week-old female SCID-beige mouse (*n* = 3) (Janvier Labs, Le Genest Saint-Isle, France) were anesthetized with 2% isoflurane (Isoflo, Esteve Farma, Carnaxide, Portugal) and kept under anaesthesia for the entire procedure. The area was prepared for sterile surgery by shaving off the fur and scrubbing with betadine solution and sterile 4 × 4 gauze. A midline ventral incision was made in the skin and the thoracic cavity. Once open, the heart was located, and an inlet needle connected to the perfusion pump was inserted into the left ventricle before puncture of the right atrium. After washing with DPBS, 10 × 10^6^ GFP-labelled MDA-MB-231 cells were circulated through the whole vascular system at 1 mL/min for 5 min. This represents a lower but comparable rate of CTC perfused in this in vivo model, in comparison to the computational and the in vitro approaches, related to the technical and ethical limitations of the model, but it permitted to achieve a relevant number of expected cellular events at the bifurcation of blood vessels to qualitatively confirm the CTC arrest in the areas of reduced flow. Afterwards, blood vessels were labelled by perfusion of Vybrant DiI cell-labelling solution (Life Technologies, Eugene, OR, USA) at 1 mL/min for 5 min. Lungs were surgically removed and fixed using PFA 4% and sliced into 1 mm thick sections for microscope examination using a LEICA DMi8 fluorescence microscope (Leica Microsystems, Wetzlar, Germany). The ARRIVE guidelines were followed to report the compliance with the quality requirements preparing the manuscript. Note that this study was performed for qualitative confirmation of the CTC arrest events, so no control untreated group was included and no randomisation or statistical approach was applied for quantification purposes. All animals were found positive for GFP-MDA-MB-231 presence in the vasculature so no animal was excluded from the study.

## Supplementary Information

Supplementary Information 1.

Supplementary Information 2.

Supplementary Information 3.

## Data Availability

All data generated or analysed during this study are included in this published article (and its Supplementary Information file).
