# Supplementary material for: Haemodynamic-dependent arrest of circulating tumour cells at large blood vessel bifurcations as new model for metastasis
Source: Sci Rep. 2021 Dec 1;11:23231. doi: 10.1038/s41598-021-02482-x (PMC8636484; doi:10.1038/s41598-021-02482-x)
Supplement: Supplementary file 1 — Supplementary Information 1. [file 41598_2021_2482_MOESM1_ESM.docx]

**Supplementary Tables and Figures**

|  | **Mesh type (90º geometry)** | | |
| --- | --- | --- | --- |
|  | **Mesh 1** | **Mesh 2** | **Mesh3** |
| **Number of cells** | 89442 | 107158 | 249718 |
| **Max. Velocity [m/s]** | 0.063142 | 0.062918 | 0.063934 |
| **Variation** | ********* | 0.35% | 1.24% |

**Table S1.** Details of different meshes used to discretize the physical domain of a 90º degrees bifurcation with a sharp angle. The values of the variables obtained for meshes 2 and 3 are compared with those obtained for mesh 1 in order to estimate the percentage of variation.

|  | **Medium** | | | **FBS** | | | **Methylcellulose 5%** | | |
| --- | --- | --- | --- | --- | --- | --- | --- | --- | --- |
|  | **Mesh 1** | **Mesh 2** | **Mesh 3** | **Mesh 1** | **Mesh 2** | **Mesh3** | **Mesh1** | **Mesh2** | **Mesh 3** |
| **Number of cells** | 35180 | 105637 | 204365 | 35180 | 105637 | 204365 | 35180 | 105637 | 204365 |
| **Residence Time [s]** | 0.2091 | 0.1995 | 0.203 | 0.2234 | 0.219 | 0.2289 | 0.2355 | 0.2439 | 0.2386 |
| **Variation** | ****** | 4.60% | 2.93% | ****** | 1.97% | 2.46% | ****** | 3.57% | 1.32% |

**Table S2.** Details of different meshes used to discretize the physical domain of 90º degrees bifurcation with a non-sharp angle.

|  | **Density [g/cm^3^]** | **Viscosity [mPa·s]** |
| --- | --- | --- |
| **Methylcellulose 0.5%** | 1.0076 | 2.29 |
| **FBS 100%** | 1.016147 | 1.935 |
| **FBS 10%** | 1.006881 | 1.49 |
| **Medium** | 1.005248 | 1.46 |

**Table S3.** Density and viscosity values of the different media used in the experiments. The same values were considered in the numerical simulations


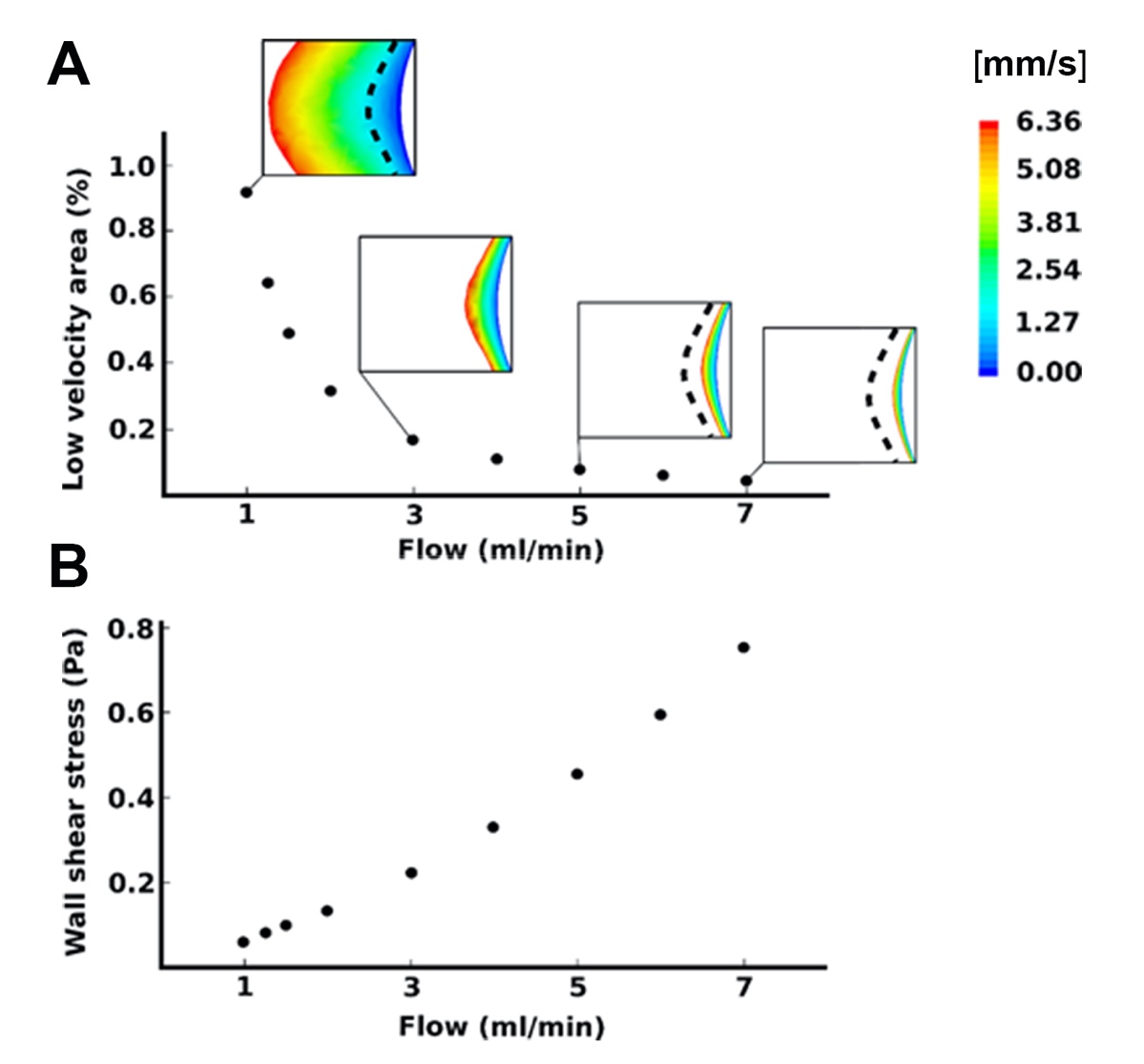


**Figure S1.** (A) Numerical representation of the low-velocity areas at the carina of the bifurcations of large vessels depending on the flow, and considering FBS as fluid in circulation. The colour scale represents the velocity of the flow in mm/s at the different areas of the bifurcation; the dashed black line marks the boundary of the low velocity area reached considering the standard flow condition (3mL/min). Similar results were observed when culture medium without FBS or with methylcellulose were used as fluid. (B) Concordant representation of the mean value of wall shear stress (WSS) analysed at the carina of the bifurcations, depending on the flow rate. Wall shear stress (WSS), as the force acting tangential to the surface of the microchannels depending on the different flow conditions, was calculated as:

$${\tau_{v}=\mu\left. \left( \frac{\partial v}{\partial r} \right) \right|}_{r=r_{v}}$$

where µ is the dynamic viscosity, v is the blood flow velocity and r is the radius of the blood vessel.


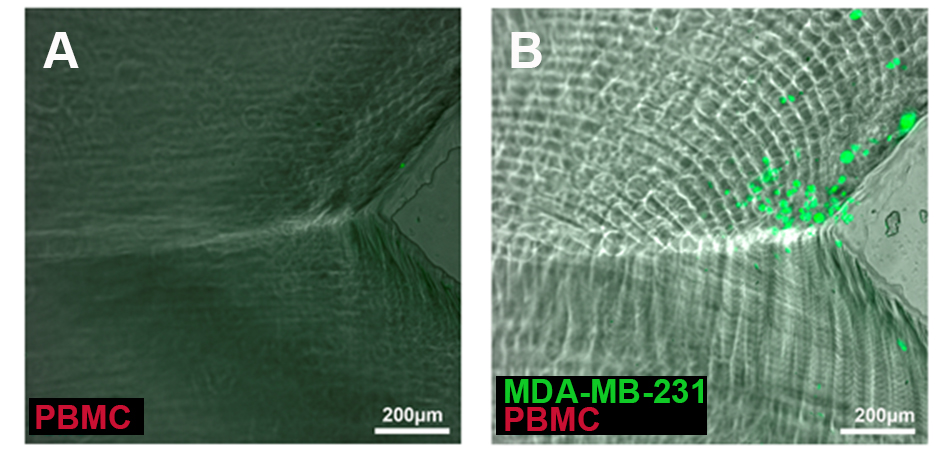


**Figure S2.** (A) Representative fluorescent image of DiD-labelled peripheral blood mononuclear cells (PBMC), perfused for two hours at 3mL/min in the microchannels; note that no arrest of PBMC was observed at the low-velocity area of the carina of the bifurcation. (B) Representative fluorescent image of GFP-MDA-MB-231 cells and DiD-labelled PBMC, after concomitant perfusion in the microchannels at 3 mL/min for two hours; note that only arrested GFP-MDA-MB-231 cells were observed at the low-velocity area of the bifurcation resulting from the inertial trajectories.


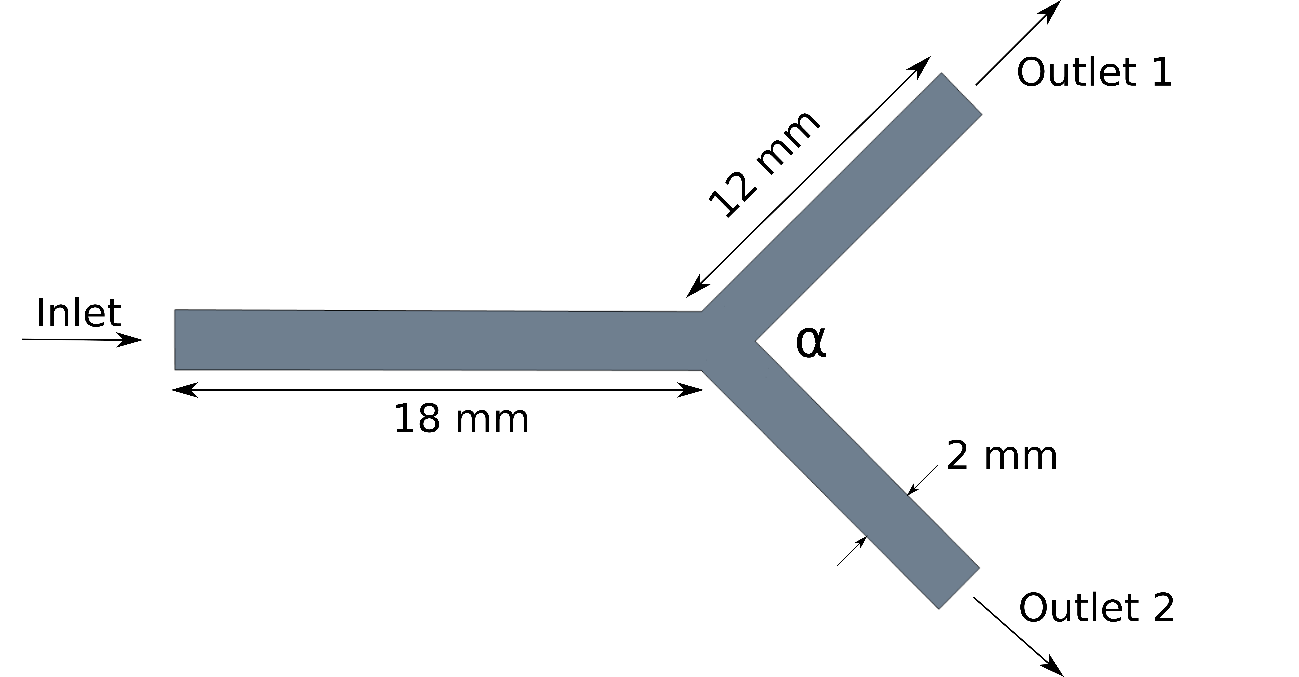


**Figure S3.** General scheme of a Y-shaped bifurcation


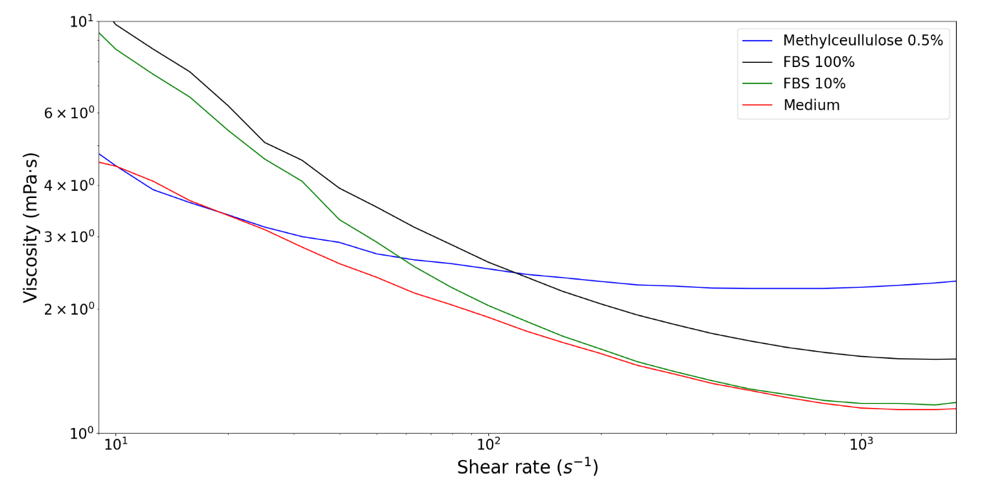


**Figure S4.** Experimental measurements of the viscosity of the fluids used as a function of the shear rate. These viscosities were measured using Anton Paar MCR 102 rheometer.


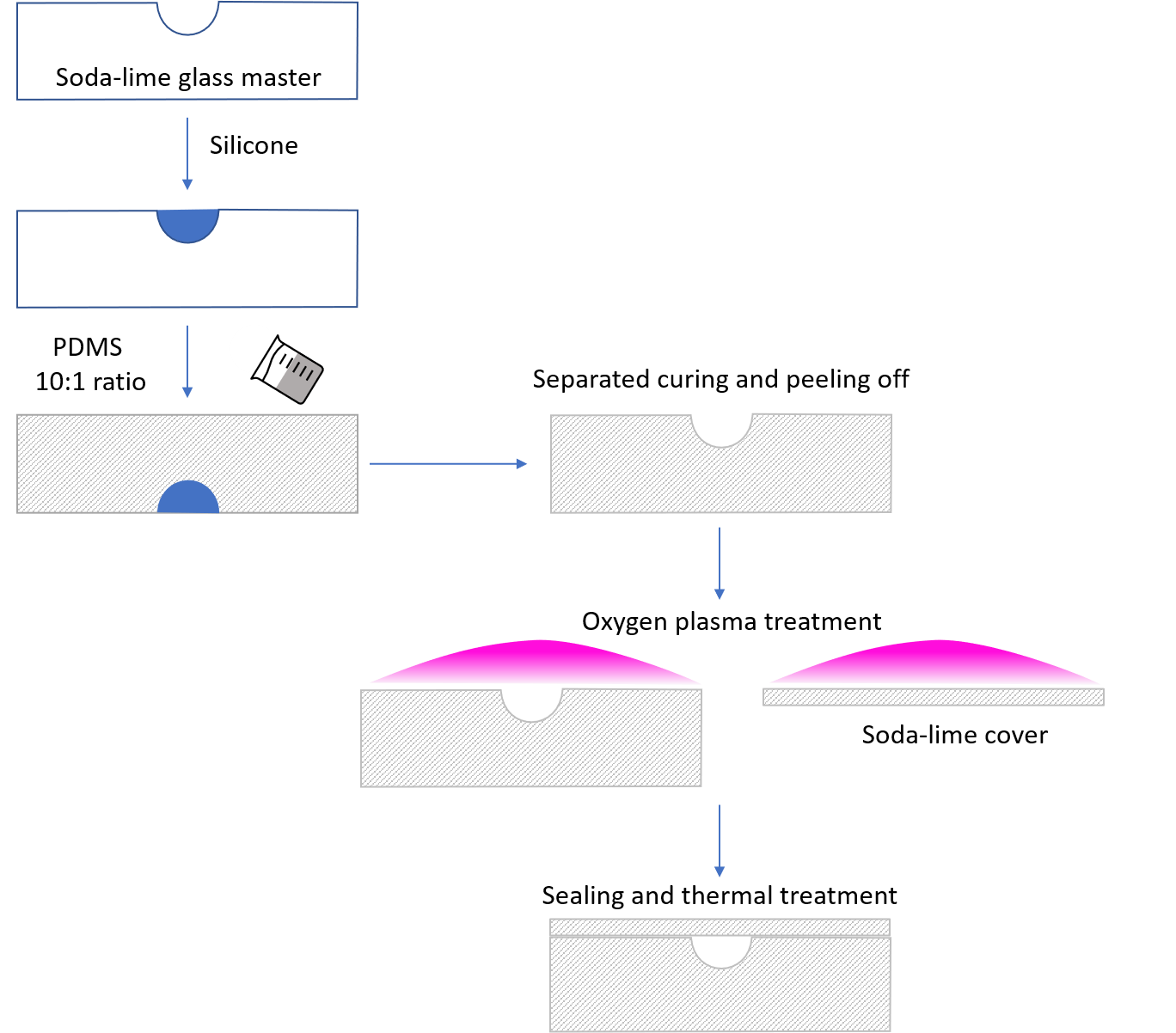


**Figure S5:** Scheme of soft-lithography process. Soda-lime glass master is fabricated with a Nd:YAG laser. An inverse silicon master was obtained. The inverse mould is covered with PDMS in a ratio 10:1. The final device is bounded by plasma cleaner technique.
